# Active Viscoelasticity of Sarcomeres

**DOI:** 10.3389/frobt.2018.00069

**Published:** 2018-06-14

**Authors:** Khoi D. Nguyen, Neelima Sharma, Madhusudhan Venkadesan

**Affiliations:** Department of Mechanical Engineering and Materials Science, Yale University, New Haven, CT, United States

**Keywords:** muscle viscoelasticity, sarcomere mechanics, active perturbation response, variable impedance, stress relaxation timescale, dynamic modulus, sinusoidal response

## Abstract

The perturbation response of muscle is important for the versatile, stable and agile control capabilities of animals. Muscle resists being stretched by developing forces in the passive tissues and in the active crossbridges. This review focuses on the active perturbation response of the sarcomere. The active response exhibits typical stress relaxation, and thus approximated by a Maxwell material that has a spring and dashpot arranged in series. The ratio of damping to stiffness in this approximation defines the relaxation timescale for dissipating stresses that are developed in the crossbridges due to external perturbations. Current understanding of sarcomeres suggests that stiffness varies nearly linearly with neural excitation, but not much is known about damping. But if both stiffness and damping have the same functional (linear or not) dependence on neural excitation, then the stress relaxation timescale cannot be varied depending on the demands of the task. This implies an unavoidable and biologically unrealistic trade-off between how freely the crossbridges can yield and dissipate stresses when stretched (injury avoidance in agile motions) vs. how long they can maintain perturbation-induced stresses and behave like a solid material (stiffness maintenance for stability). We hypothesize that muscle circumvents this trade-off by varying damping in a nonlinear manner with neural excitation, unlike stiffness that varies linearly. Testing this hypothesis requires new experimental and mathematical characterization of muscle mechanics, and also identifies new design goals for robotic actuators.

## 1. Introduction

**Figure 1:**
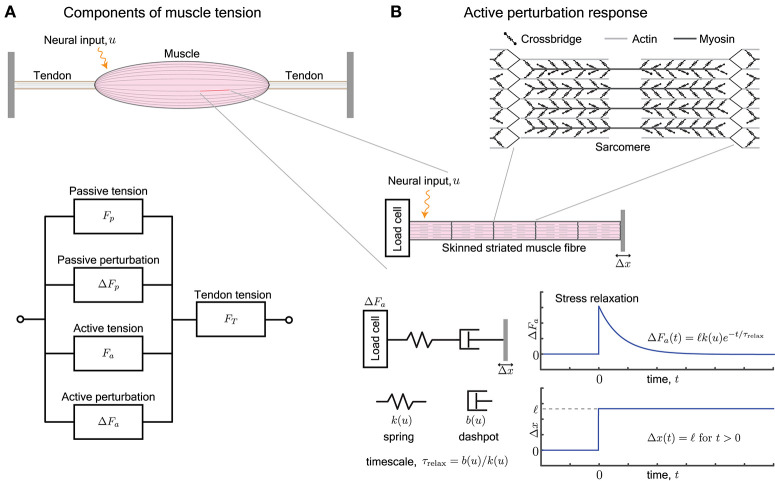
**(A)** A muscle provides, at every length, a passive tension *F*_*p*_ and an active tension *F*_*a*_ that varies with neural input. Furthermore, an external perturbation to its length elicits additional passive and active contributions, Δ*F*_*p*_ and Δ*F*_*a*_, respectively. The tendons attached to the muscle work in series and act to transmit the total tension *F*_*T*_ the muscle outputs, i.e., *F*_*T*_ = *F*_*p*_ + Δ*F*_*p*_ + *F*_*a*_ + Δ*F*_*a*_. **(B)** The active perturbation response arises from the contractile machinery of sarcomeres, and can be measured by applying length perturbations Δ*x* to an isolated skinned muscle fiber. The perturbation response Δ*F*_*a*_ is approximated by a Maxwell-type material that is comprised of a spring and dashpot in series. The spring has stiffness *k*(*u*) and dashpot has damping *b*(*u*), both of which vary with the neural input *u*. The ratio of the damping to stiffness determine a stress relaxation timescale τ_relax_. For a step length perturbation, the active perturbation response Δ*F*_*a*_(*t*) decays exponentially over the relaxation time.

A muscle develops mechanical forces when neurally or electrically excited, and also when externally perturbed (Rack and Westbury, 1974; Kirsch et al., 1994; Lindstedt and Hoppeler, 2016). The perturbation response Δ*F*_*p*_ of passive tissues and the active excitation-dependent perturbation response Δ*F*_*a*_ add to the baseline force *F*_*a*_ generated due to neural excitations and to *F*_*p*_ due to passive tissues (Figure 1A). Passive refers to mechanical responses in the absence of neural stimulation while active responses require neural stimulation, and consume metabolic energy. Notably, the active resistance to stretch is one of the first mechanical responses of muscle when stimulated, even before it begins to develop tension (McMahon, 1984).

Active and passive perturbation responses play an essential role in animal motor control because they are faster than any neural response, including the fastest of reflexes (Bizzi et al., 1982; Brown and Loeb, 2000; Dickinson et al., 2000; Hogan and Buerger, 2005; Holmes et al., 2006; Nishikawa et al., 2007; Biewener, 2016; Roberts, 2016). These responses have also been called preflexes or mechanical feedback (Brown and Loeb, 2000; Nishikawa et al., 2007). In robotics as well, the perturbation response of actuators are employed advantageously when appropriately tuned to the task and the environment's mechanical response (Hogan, 1984; Pratt and Williamson, 1995; Hogan and Buerger, 2005; Buerger and Hogan, 2007; Vanderborght et al., 2013). However, current actuator technologies do not yet match the ability of muscle to vary its active perturbation response by several fold, such as stiffness that may vary by over 50× in muscle (Hunter and Lafontaine, 1992; Madden, 2007; Anderson et al., 2012; Vanderborght et al., 2013; Hines et al., 2017). Therefore the mechanical capabilities of skeletal muscle continues to be sought-after by roboticists not only in terms of their force, work and power generation capabilities (Madden, 2007), but also in terms of their active perturbation response (Madden et al., 2004).

In this review, we examine the active perturbation response using the formalism of frequency-dependent dynamic modulus, i.e., the ratio of the force response Δ*F*_*a*_ to externally imposed small sinusoidal length perturbations Δ*x* (Figure 1B). Emphasizing qualitative over detailed quantitative comparisons with data, we use approaches from materials science (de Gennes, 1979; Barnes et al., 1989) to simplify the possibly complicated active response in terms of spring-like and dashpot-like elements (Figure 1B). In particular, because the active perturbation response of muscle does not have a single, simple rest length and decays with time, its simplest representation is a Maxwell material with a spring and dashpot in series (Maxwell, 1867; Barnes et al., 1989) whose stiffness *k* and damping *b* depend on the neural input *u*.

We use examples from motor control in section 2 to illustrate the role of active perturbation responses, and present an overview in section 3 of how a muscle's active perturbation response is characterized. We elaborate the Maxwell model in section 4, examine its neural modulation in section 5, and summarize the main conclusions of this review in section 6.

## 2. Active perturbation response in motor control

Active perturbation response of muscle depends strongly on the animal's intent and biomechanical context. At one extreme, muscle may behave like a stiff solid that undergoes no appreciable strain (e.g., co-contracted muscles to stiffen a joint). At another extreme it may offer little resistance and yield freely like a fluid (e.g., biceps in throwing). In general, muscles exhibit every behavior in between the extremes depending on the level of neural excitation (Cui et al., 2008; Sponberg and Full, 2008; Farahat and Herr, 2010; Hu et al., 2011; Sawicki et al., 2015). Consider three representative examples with different active perturbation responses of muscles and their analogs in robots.

First, when elastic energy storage and recovery are important, a muscle typically behaves as a stiff strut (Alexander and Bennet-Clark, 1977; Zajac, 1989; Biewener and Roberts, 2000; Roberts, 2016) so that most of the externally imposed strain is elastically stored in and recovered from the tendon (but see George et al., 2013). Use of the tendon's series elasticity to store and recover elastic energy in running is a well-known example (Cavagna et al., 1964). Robotic actuators employ a similar approach using series elastic elements for energy storage and recovery, in addition to protecting the actuator from shock loads (Raibert, 1986; Pratt and Williamson, 1995).

Second, when stabilizing a joint through muscle stiffening or regulating limb stiffness for controlling interactions with the surroundings, the stiffness of muscle is more continuously and appropriately varied to the dynamics and mechanics of the task being performed (Lacquaniti and Maioli, 1987; Lacquaniti et al., 1993; McIntyre et al., 1996; Burdet et al., 2001; Hogan and Buerger, 2005; Cui et al., 2008; Hu et al., 2011). In dynamical contexts, muscle's active perturbation response is generalized to an active impedance (Hogan, 1984; Hogan and Buerger, 2005) that may be approximately understood in terms of spring-like and dashpot-like responses to the external strain, strain-rate and the neural excitation. Varying limb impedance by modulating the impedance of the driving actuators has also been central to controlling interactions in robots (Hogan and Buerger, 2005; Vanderborght et al., 2013).

Third, some tasks involve quick transitions of muscles from a fluid-like to a solid-like response. For example, in throwing, the elbow's joint angular velocity exceeds 5000°/s before the elbow rapidly brakes and stiffens at the end to avoid injuries (Roach et al., 2013). Antagonistic muscles to this movement, such as the *biceps brachii* have to yield with little resistance like a weak dashpot would, in order to not prematurely decelerate the arm and to avoid injuries when a rapid stretch is imposed upon them (Lindstedt et al., 2001; LaStayo et al., 2003). At the end of the motion, the *biceps brachii* provide active braking to safely decelerate the arm without themselves suffering damage, thus transitioning into impedance control and ending with high stiffness. Such transitions in the material properties of actuators have not been studied or used in robots.

These examples illustrate that muscle's active perturbation response bridges the gap between two extremes. One extreme is the ability to maintain internal stresses that arise from an external perturbation for prolonged periods of time, lasting several minutes (Rancourt and Hogan, 2001; Loram et al., 2007), so that the muscle may function as a solid spring-like material for stability, elastic energy storage in tendons and so on. The other extreme is the ability to rapidly dissipate perturbation-induced internal stresses in mere tens of milliseconds (Roach et al., 2013) so that the muscle may function as a fluid dashpot-like material that enables agile and rapid motions without suffering damage.

## 3. Characterization of muscle's active perturbation response

Current understanding of muscle's mechanical behavior may be encapsulated by simple mathematical models such as Hill-type models of muscles (Zajac, 1989), Huxley-type models of sarcomeres (Walcott, 2014), and more intricate models of non-crossbridge elements such as the winding filament model of titin (Nishikawa et al., 2012; LeMoyne et al., 2014). Such simplified models are essential to elucidate underlying biological principles (Herzog, 2000), and to facilitate intensive computations such as applications of optimal control to study motor behaviors (Todorov, 2004). These mechanical models used to understand and characterize muscle have clear analogs in the centuries-old development of constitutive models of viscoelasticity in materials science (Barnes et al., 1989). An important lesson from materials science (Barnes et al., 1989) and from recent developments in soft (Wyss et al., 2007; Goldenfeld, 2018) and active or biological matter (Mizuno et al., 2007; Marchetti et al., 2013) has been that simple models, although often quantitatively inaccurate, guide experimental design and form the basis for fundamental mechanistic understanding.

Among viscoelastic constitutive models of materials, the Voigt model with a spring and dashpot in parallel, and the Maxwell model with a spring and dashpot in series are the two simplest approximations (Barnes et al., 1989). The Voigt model has been applied to characterize the viscoelastic properties of passive tissues, including that of muscle (Fung, 2013). But it is qualitatively wrong for the active perturbation response of muscle because it implies a single fixed length and the persistence of elastic stresses forever. We therefore consider the Maxwell model (Figure 1B) or a generalization called the standard linear model, which is a combination of a Maxwell body in parallel with a second weak spring representing parallel passive elasticity. These models are not literal representations of microscopic springs and dashpots, but their stiffness and damping are the respective linearized parameterizations of the reversible (elastic) and irreversible (viscous) components of the dynamic response to perturbations. In this sense, they are applicable to passive biological materials (Fung, 2013), as well as to the perturbation response of active biological materials (Deng et al., 2006; Mizuno et al., 2007).

We briefly summarize how muscle generates forces, with special focus on how it resists perturbations, and point the reader to more thorough examinations of the century-old topic of force generation in muscle (Herzog, 2000). Neural excitation in the form of a train of action potentials increases the concentration of freely available intracellular Ca^2+^ ions, and in turn increases the number of crossbridges formed between interdigitating filaments of actin and myosin (Figure 1B). The crossbridges form transient load-bearing links between actomyosin filaments. By executing a power stroke, each crossbridge contributes approximately 2pN to the active tension *F*_*a*_. It also acts as a molecular spring with a stiffness of approximately 1pN/nm and contributes to the active perturbation response Δ*F*_*a*_ (Finer et al., 1994). The collective behavior of crossbridges is such that they store elastic stresses when the sarcomere is externally perturbed, but slowly dissipate the stresses as crossbridges detach and re-attach elsewhere (Huxley, 1974). The presence of a relaxation timescale for stress dissipation suggests that the active perturbation response resembles a Maxwell viscoelastic material (Palmer et al., 2007).

### 3.1. Dynamic modulus and other measures of perturbation response

The dynamic modulus is the ratio of the active perturbation response Δ*F*_*a*_ to externally applied small stretches Δ*x*, and depends on the excitation level (or intracellular [Ca^2+^]) and the rate (frequency) at which the stretch is applied. The behavior resembles a pure spring if the stresses Δ*F*_*a*_ persist without any decay, and resembles a pure dashpot if the stresses decay (exponentially for a linear dashpot). Most materials exhibit both behaviors, depending on the timescales of the applied stretch and of observation. For example, a Maxwell body responds to a sudden stretch with a sharp pure spring-like transient, followed by a slower pure dashpot-like dissipation of stress (Figure 1B). These responses to small perturbations have also been characterized and identified as the short-range elastic component (SREC) (Rack and Westbury, 1974; Campbell, 2010), the complex modulus, or the sinusoidal response (Kawai and Brandt, 1980; Palmer et al., 2007).

The dynamic modulus arising from crossbridge dynamics is not the same as the slopes of the well-known force-length and force-velocity curves of the sarcomere (Zajac, 1989), which are sometimes misinterpreted as stiffness and damping. Although the slope of these respective curves have physical units of stiffness and damping, they do not represent the dynamic perturbation response of the sarcomere. For example, the force-length curve for a sarcomere near its natural length of ≈ 2.2μm has zero slope, which leads to a misinterpretation that an excited sarcomere has no stiffness at its natural length, a provable fallacy (Rack and Westbury, 1974). Seen from the perspective of dynamic systems, the sarcomere may be characterized as a material with a frequency-dependent perturbation response, and the force-length relationship quantifies the zero-frequency stiffness alone (Kawai and Brandt, 1980). It is tempting to set aside these complicated (and complex) perturbation responses of muscle as biological artifacts. However, as we argue here, the frequency-dependent perturbation response of muscle, and its modulation through neural excitation, are central to muscle's utility as a biological actuator.

The perturbation response of muscle has also been extensively used as a window into its microscopic functioning (McMahon, 1984). Among the many insights gleaned on sarcomere function, there remain major open questions such as the molecular origins of force enhancement (Rassier, 2012) and thixotropy (Campbell, 2010). Force enhancement (or depression) is the persistence of additional stresses for several seconds when an active sarcomere is externally stretched (contracted Edman et al., 1982). Thixotropy is a term borrowed from passive materials to refer to the history dependence of a cyclically stretched sarcomere so long as the time elapsed between consecutive stretches is sufficiently small (Campbell and Moss, 2002). These and similar studies have revealed that besides the actomyosin contractile machinery, muscle's force generation and perturbation response are additionally affected by other factors such as the surrounding passive tissues (Roberts, 2016), pressure and viscosity of the intra- and inter-cellular fluid (Baron et al., 2017; Sleboda and Roberts, 2017), and non-crossbridge but calcium-sensitive components like titin (Herzog, 2014; Nishikawa, 2016). Frequency-dependent dynamic modulus is related to the perturbation response protocols used in force enhancement and thixotropy studies, but generalizes to an examination of multiple timescales by adopting established protocols from rheology.

The frequency-dependent dynamic modulus of a sarcomere, myofibril, muscle fiber or a whole muscle is mostly attributable to the crossbridges only when the imposed stretches are sufficiently small so as to not forcibly detach bound crossbridges (McMahon, 1984). A natural concern therefore is whether the dynamic modulus is relevant for animal motor behaviors. Forcibly detaching crossbridges leads to microscopic damage that helps build muscle if the extent of damage is sufficiently small (LaStayo et al., 2003), but excessive damage is injurious. Thus when muscle is highly externally stretched, it is important for the sarcomere itself to remain stiff and enable the softer series elastic elements such as the tendon, the aponeuroses, and other passive tissues to accommodate a majority of the strain. In muscles with short tendons or when forcibly stretched by amounts that cannot be accommodated by tendons, the crossbridges must unbind and dissipate stresses fast enough so that a majority are not forcibly detached. Thus non-injurious perturbation response involves small elastic strains at individual crossbridges although the whole muscle or joint may experience large motions, and large crossbridge strains imply dashpot-like stress dissipation. We therefore argue that the small strain dynamic modulus of the muscle is relevant to non-pathological function, and is indeed known to play a role in a large variety of tasks (Bizzi et al., 1982; Lacquaniti and Maioli, 1987; Lin and Rymer, 1998, 2000, 2001; Loram et al., 2007; Cui et al., 2008; Rancourt and Hogan, 2009; Hu et al., 2011).

## 4. Active perturbation response of a maxwell material

A Maxwell-type response is modeled by a series arrangement of a spring with stiffness *k*(*u*) and dashpot with damping *b*(*u*), and captures the relaxation behavior of the active perturbation response Δ*F*_*a*_ (Figure 1B). The dependence on neural input *u* represents the fact that the number of crossbridges and therefore the active response of a sarcomere varies with neural excitation (Herzog, 2000). Upon imposing a step perturbation to the length, the spring initially assumes all the externally applied strain and Δ*F*_*a*_ = ℓ*k*(*u*) where ℓ is the amplitude of the imposed step. The dashpot dissipates the stored elastic stress over a timescale τ_relax_ = *b*(*u*)/*k*(*u*) and Δ*F*_*a*_ decays exponentially in time. The response resembles an elastic solid against perturbations completed over a duration shorter than the relaxation timescale τ_relax_ and a viscous fluid against longer perturbations.

The perturbation response of a Maxwell material is characterized by its frequency-dependent dynamic modulus *K*. The imposed length perturbation Δ*x*(*t*) is distributed between the displacement Δ*x*_*s*_ of the spring and Δ*x*_*d*_ of the dashpot so that Δ*x* = Δ*x*_*s*_ + Δ*x*_*d*_. Using the constitutive laws for a spring and dashpot, the active perturbation response is given by

$$\begin{array}{r} \frac{d}{dt}\Delta x=\frac{1}{k\left(u\right)}\frac{d}{dt}\Delta F_{a}+\frac{1}{b\left(u\right)}\Delta F_{a}. \end{array}$$

The normalized dynamic modulus *K*/*k*(*u*) is found using the Fourier transform of Equation (1), and depends on the frequency ω of the applied sinusoidal length perturbation and the stress relaxation timescale τ_relax_ according to

$$\begin{array}{rcl} \frac{K}{k\left(u\right)} & = & |\frac{\hat{f}}{\hat{x}}|=\frac{\omega \tau_{\text{relax}}}{\sqrt{1+{\left(\omega \tau_{\text{relax}}\right)}^{2}}}, \end{array}$$

$$\begin{array}{rcl} \tau_{\text{relax}}\left(u\right) & = & \frac{b\left(u\right)}{k\left(u\right)}, \end{array}$$

where $\hat{x}\left(\omega \right)$ and $\hat{f}\left(\omega \right)$ are the Fourier transforms of Δ*x*(*t*) and Δ*F*_*a*_(*t*), respectively. For fast perturbations with ωτ_relax_ ≫ 1, the dynamic modulus *K* ≈ *k*(*u*), and the active perturbation response resembles a pure spring. For slower perturbations with ωτ_relax_ ≪ 1, the dynamic modulus *K* ≈ τ_relax_*k*(*u*)ω and decreases linearly with frequency. The prefactor τ_relax_*k*(*u*) is equal to damping *b*(*u*), and the perturbation response resembles a pure dashpot.

**Figure 2:**
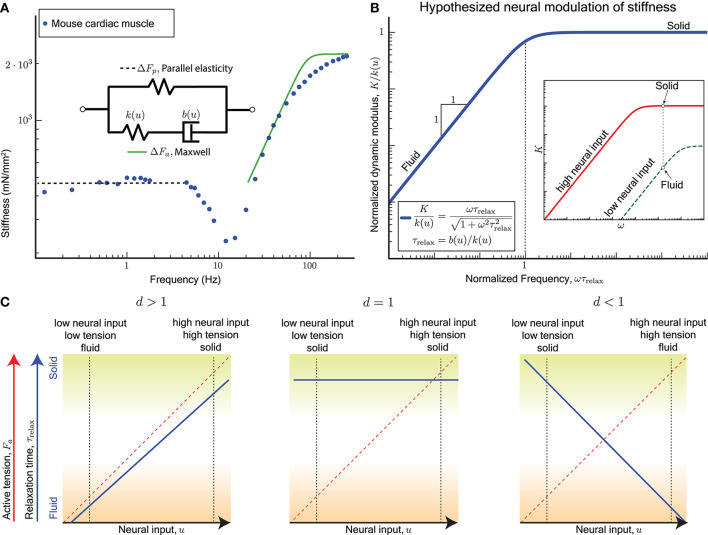
**(A)** Sinusoidal analysis of skinned mouse cardiac muscle fibers shows an active perturbation response qualitatively similar to a Maxwell-type material for frequencies above 10 Hz. A parallel elastic response dominates at lower frequencies, and the active component is no longer evident. Data replotted from Palmer et al. (2007). **(B)** plot: The viscoelastic perturbation response for a Maxwell-type material where τ_relax_ is the timescale of stress relaxation, *K* is the dynamic modulus of the muscle, and ω is the frequency of a sinusoidal perturbation. Against perturbations with periods shorter than τ_relax_, the response resembles an elastic solid, and it resembles a viscous fluid for slower perturbations. inset: If the relaxation time for the active perturbation response of a sarcomere depends on its neural input *u*, we hypothesize that the sarcomere may switch between solid and fluid behaves by varying *u*. For example, the active perturbation response may be fluid-like at low neural input (green) and solid-like at high neural input (red). **(C)** For the hypothesized dependence $\tau_{\text{relax}}\propto u^{d-1}$ (Equation 6), τ_relax_ is increasing with increasing *u* if *d* > 1, invariant with *u* if *d* = 1, and decreasing with increasing *u* if *d* < 1. Each case results in different functional consequences for a sarcomere and ultimately for muscles when coupled with the fact that a sarcomere's active tension *F*_*a*_ increases with *u*. A sarcomere can vary between a solid behavior with high active tension (mechanically stable against external perturbations, green shading) and a fluid behavior with low active tension (freely yields with minimal resistance, brown shading) only if *d* > 1.

The active perturbation response of skinned muscle fibers (Kawai and Brandt, 1980; Palmer et al., 2007) indeed resembles a Maxwell material's active response Δ*F*_*a*_ plus a weak passive parallel elastic spring Δ*F*_*p*_ (Figure 2A). The dynamic modulus measurement of skinned cardiac muscle fibers using sinusoidal perturbations of different frequencies is shown in Figure 2A, where activated fibers were held at the plateau region of the force-length curve and perturbed sinusoidally. Data from skeletal muscle (Kawai and Brandt, 1980; Miller et al., 2010; Palmer et al., 2013) show a similar response but those experiments did not perturb at high enough frequencies, because of which the dashpot-like response is evident but not the spring-like response.

Cardiac muscles have many physiological differences from skeletal muscles, but both are governed by similar biomechanical principles by virtue of relying on sarcomeres for active force production. Whether the Maxwell body is attributed to crossbridge dynamics, titin, or other active components of the sarcomere, the sinusoidal analysis experiments indicate that a single timescale, namely the stress relaxation timescale τ_relax_, separates the fluid and solid behaviors of the active perturbation response.

A non-dimensional version of the Maxwell model has no parameters and is obtained by normalizing the dynamic modulus *K* by the excitation-dependent high-frequency stiffness *k*(*u*), and normalizing the frequency ω by the reciprocal of the stress relaxation timescale τ_relax_ (Figure 2B). Therefore if the relaxation timescale τ_relax_ were made excitation-dependent so that the corner of the frequency response shifts right at lower excitation levels, it would also reduce the high frequency stiffness (red vs. green curves in Figure 2B inset). Thus, at the same frequency of perturbation, the active response may transition from a stiff solid to a weakly visous fluid if the excitation is sufficiently reduced.

## 5. Varying the relaxation timescale

The stress relaxation timescale τ_relax_ of the active perturbation response is the ratio of the sarcomere's damping to its stiffness, and may vary with neural excitation depending on how stiffness and damping vary (Figure 2B). While it is known that the stiffness increases linearly with neural input (Rack and Westbury, 1974; Kirsch et al., 1994), it remains unknown how damping may vary. To examine the neural modulation of τ_relax_, we consider a linear form for the stiffness *k*(*u*), but allow a general power-law for how damping *b*(*u*) varies with neural input according to

$$\begin{array}{rcl} k\left(u\right) & = & k_{0}u, \end{array}$$

$$\begin{array}{rcl} b\left(u\right) & = & b_{0}u^{d}. \end{array}$$

Thus the stress relaxation timescale is given by the ratio of damping to stiffness as

$$\begin{array}{r} \tau_{\text{relax}}=\tau_{0}u^{d-1}. \end{array}$$

Neural excitation is parameterized by the normalized variable 0 ≤ *u* ≤ 1, and τ_0_ = *b*_0_/*k*_0_ is the relaxation timescale at maximal excitation.

Recall that evidence from motor control (section 2) indicates that the stress relaxation timescale increases with increasing neural drive. Highly activated muscle resembles an elastic solid whose relaxation timescale is much greater than the duration of the experiment or behavior (green shading in Figure 2C), and minimally activated muscle resembles a weakly viscous fluid whose relaxation timescale is much smaller than the fastest perturbation in the experiment or behavior (brown shading in Figure 2C).

Only for *d* > 1 is the qualitatively correct behavior observed for τ_relax_ as a function of *u* (blue solid line, left panel of Figure 2C), and if both stiffness and damping scale equally with excitation (*d* = 1) the timescale becomes an invariant quantity (blue solid line, middle panel Figure 2C). For *d* < 1 the modulation of τ_relax_ with increasing *u* makes the active perturbation response weak spring-like for weak neural input and strong dashpot-like for strong neural input (blue solid line, right panel Figure 2C). This case would correspond to large crossbridge strains and frequent forced detachments of crossbridges at both extremes of muscle function, solid or fluid.

In all three cases, the mean active tension *F*_*a*_ would increase with the excitation *u* (red dashed line, Figure 2C), and therefore the dependence of the relaxation timescale τ_relax_ on the neural input *u* is equivalent to being dependent on the mean active tension *F*_*a*_. The functionally desirable behavior is therefore one of a force-dependent slowing of stress relaxation in the active perturbation response.

Hill-type muscle-tendon models that incorporate a series elastic tendon, and treat the force-length and force-velocity curves as the stiffness and damping of the active perturbation response, exhibit a similar frequency response to the Maxwell model (Figures 16, 18, and 19 in Zajac, 1989). The high-pass filtering characteristics of those models arise because the dynamic modulus of the active element is effectively zero and it behaves as a pure damper in response to perturbations. Thus the active response plus the passive elastic spring of the tendon is simply a Maxwell model. Furthermore, when the Hill-type models operate on the ascending limb of the force-length curve, the zero-frequency stiffness of the active response is non-zero and the stress relaxation timescale (corner in the frequency response) becomes excitation-dependent. However, the functional dependence of the stress relaxation timescale on the excitation in the Hill-type models corresponds to the unrealistic *d* < 1 scenario, where increasing excitation makes the muscle-tendon more fluid-like. This increased fluid-like response may however be relevant in dynamic tasks such as locomotion, where submaximally activated muscle operates primarily on the ascending limb of the force-length curve (Holt and Azizi, 2016).

The active perturbation response of muscle is also modulated through neural feedback circuits, the stretch reflex being the fastest of them (McMahon, 1984). Even those fast reflexes take over 50ms in humans and therefore it can impact the perturbation response only at frequencies 20Hz or below. At these lower frequencies, reflexes have the capability of altering the frequency response, including the effective damping (Lin and Rymer, 1998, 2001). The analysis presented in this paper does not incorporate the effect of neural reflexes, and neither do the single fiber experiments using sinusoidal analysis (Kawai and Brandt, 1980; Palmer et al., 2007, 2013; Miller et al., 2010).

## 6. Conclusion

We hypothesize that an excitation-dependent increase in the damping associated with the active perturbation response should outstrip the stiffness increase. This is necessary to explain observed muscle behavior and accomplish tasks that require the muscle to mimic phase transitions between a fluid-like and solid-like active response. The microscopic origins of how the stress relaxation timescale may vary is an important avenue for future research. Whether it shares mechanisms with force-enhancement, thixotropy or even the well-known load-dependent changes in the detachment rate of actin-bound myosin remains to be discovered. Our proposal for studying the active perturbation response of sarcomeres has analogies to the applications of rheology in the fields of active and biological matter (Deng et al., 2006; Mizuno et al., 2007; Wyss et al., 2007; Marchetti et al., 2013).

Based on the known uses of muscle in control, and current theories of sarcomere function, we have argued for treating the active perturbation response of the sarcomere as a Maxwell-type material with the elastic and viscous elements in series. We note that our conclusion differs from some authors who express a preference for a Voigt element, although they conclude that both models could fit their experimental data (Ford et al., 1977). A significant reason for the differing viewpoints is because we explicitly separate the active perturbation response from all other force production components, namely the passive perturbation response, and the active and passive mean force generation, while (Ford et al., 1977) have lumped them together.

A renewed examination of the active perturbation response of muscle and its control through neural excitation may provide new design targets for engineered actuators for use in agile animal-like robots. However, significant technological challenges remain in developing actuators that can modulate stiffness and damping. The prevalent use of high-bandwidth feedback control to mimic viscoelasticity creates fragile devices that are sensitive to sensor malfunction, time-delays and noise (Hogan and Buerger, 2005). On the other hand, novel soft actuators (Majidi, 2014; Rus and Tolley, 2015; Hines et al., 2017) such as dielectric (Anderson et al., 2012) or nematic (Hines et al., 2017) elastomers, twisting cable muscles (Haines et al., 2016) or pneumatic actuators (Wehner et al., 2014; Peele et al., 2015) resemble a Voigt material rather than a Maxwell material. Therefore, they suffer the severe limitations imposed by a fixed neutral length and a strong parallel elastic component. As an alternative, variable stiffness and damping capabilities may be achieved through mechanical design of a transmission element (Vanderborght et al., 2013). These designs also have a key limitation, namely that force, stiffness and damping are typically varied through independent control inputs and thereby lead to high control complexity. We do not imply that matching the success of muscles as actuators needs mimicry of its microscopic structure. Rather, developing novel actuators with a Maxwell-type perturbation response that can undergo large changes in the stress relaxation timescale may prove fruitful in mimicking the beneficial principles underlying muscle's versatile use by animals.

## Author contributions

KN and NS contributed equally. MV conceived the paper. KN synthesized the literature on sarcomere mechanics. NS synthesized the motor control literature. KN, NS, and MV wrote and edited the paper together.

### Conflict of interest statement

The authors declare that the research was conducted in the absence of any commercial or financial relationships that could be construed as a potential conflict of interest.
